# Sarcopenic obesity in older adults: a scoping review of prevalence, assessment tools, associated factors, and interventions

**DOI:** 10.3389/fpubh.2026.1792543

**Published:** 2026-03-24

**Authors:** Yihua Ding, Yanhong Zhang, Yaqin Zhu, Yuxuan Qin, Xiang Li

**Affiliations:** 1 School of Nursing, Chengde Medical University, Chengde, Hebei, China; 2 Department of Nursing, Affiliated Hospital of Chengde Medical University, Chengde, Hebei, China; 3 Department of Neurology, Affiliated Hospital of Chengde Medical University, Chengde, Hebei, China

**Keywords:** sarcopenic obesity, aged, assessment tools, influencing factors, intervention measures, scoping review

## Abstract

**Background:**

With the accelerating global population aging, the prevalence of sarcopenic obesity (SO) in older adults has been increasing year by year, which has become a major public health issue that seriously impacts the quality of life and threatens the health status of the older population.

**Objective:**

The review aimed to summarize the current status, assessment tools, influencing factors, and intervention measures related to SO among older adults, identify research gaps in the existing literature and provide a reference for future studies.

**Methods:**

The review was conducted in accordance with the Preferred Reporting Items for Systematic reviews and Meta-Analyses extension for Scoping Reviews (PRISMA-ScR) framework. Literature on the current prevalence, assessment tools, influencing factors, and interventions for older adults with SO was retrieved from the following databases: China National Knowledge Infrastructure (CNKI), Wanfang Database, PubMed, Web of Science, Cumulative Index to Nursing and Allied Health Literature (CINAHL), and Cochrane Library. The search timeframe was restricted from database inception to October 2025. Two researchers independently screened the research literature, extracted data, and summarized findings.

**Results:**

Forty-five studies were included, which were published between 2016 and 2025, with a marked increase in publications from 2021 to 2025. The prevalence of SO among older adults ranged from 2.52 to 34.62%, with notable variations across studies. Assessment tools were diverse, but a standardized evaluation framework was lacking. Influencing factors were categorized into four groups: sociodemographic factors, disease-related factors, psychosocial and behavioral factors, and nutritional factors. Intervention measures included exercise interventions, nutritional interventions, and multidomain interventions.

**Conclusion:**

The prevalence of SO among older adults varies considerably, and existing assessment tools lack a standardized framework and diagnostic thresholds that are not population-specific. Future efforts should integrate the characteristics of sarcopenia and obesity to develop predictive assessment tools specifically tailored for older adults with SO, while also focusing on the factors influencing SO to further refine intervention measures, aiming to slow disease progression and promote healthy aging.

## 1 Introduction

The global population is undergoing rapid aging, with the proportion of individuals over 60 years increasing from 12% in 2015 to 22% by 2050 (1). China is also expected to enter a stage of advanced population aging by 2030. As the older population in China continues to grow, health issues among older adults have become increasingly prominent, with the rising prevalence of both sarcopenia and obesity. It is estimated that by 2030, the prevalence of sarcopenia among individuals aged 60 years and older in China will reach 16.2% (2), while the proportion of obesity will increase to 30% (3). In older adults, sarcopenia and obesity frequently coexist and may exert synergistic effects, imposing a dual burden on the body. Based on this phenomenon, Baumgartner (4) first defined the coexistence of sarcopenia and obesity as sarcopenic obesity (SO) in 2000, sarcopenic obesity (SO) is a clinical functional condition characterized by the coexistence of obesity and sarcopenia, referring to an obese state accompanied by low skeletal muscle mass, strength, and/or physical function. Epidemiological evidence suggests that the global prevalence of SO among older adults is approximately 11% (5). Compared with sarcopenia or obesity alone, SO is associated with a higher risk of adverse outcomes, including cardiovascular disease, arthritis, and metabolic disorders, and has also been closely linked to increased mortality (6).

In 2022, the European Society for Clinical Nutrition and Metabolism (ESPEN) and the European Association for the Study of Obesity (EASO) jointly published the first international consensus definition and diagnostic algorithm for sarcopenic obesity (7). The ESPEN–EASO consensus defines sarcopenic obesity as the coexistence of excess adiposity and low muscle mass and/or muscle function. It proposes a two-step diagnostic pathway including screening and confirmation stages. This consensus was developed to harmonize diagnostic criteria and improve comparability across studies of prevalence, risk factors, and outcomes. However, despite the availability of this standardized framework, substantial heterogeneity in operational definitions and diagnostic components persists in the literature. Therefore, examining how existing studies define sarcopenic obesity in relation to the ESPEN–EASO consensus is essential for understanding the sources of variability in reported epidemiological estimates.

Currently, SO has increasingly become a focus of academic research, with existing studies mainly addressing screening and diagnosis, pathophysiological mechanisms, adverse outcomes, and interventions related to SO (8). However, the available evidence remains fragmented and lacks systematic synthesis and integration. A scoping review can not only effectively map and summarize the existing body of evidence but also identify research gaps and potential directions for future studies. Therefore, this study aims to systematically review and analyze the current research literature on older adults with SO, with particular attention to its prevalence, assessment tools, influencing factors, and intervention measures, to provide evidence-based references for healthcare professionals to improve the identification, assessment, and management of SO in clinical practice.

## 2 Methods

This review adopted the scoping review framework proposed by Arksey and O'Malley (9) to examine the prevalence, assessment tools, influencing factors, and intervention measures related to SO among older adults. This framework is particularly useful for mapping existing evidence and identifying research gaps in areas where the research literature remains fragmented. The review process adhered to the Preferred Reporting System for Meta-Analysis for Scoping reviews (PRISMA-ScR) (10). This scoping review was preregistered on Open Science Framework (OSF) Registries: https://doi.org/10.17605/OSF.IO/RDZGA.

### 2.1 Identifying research questions

Subsequent to a review of the literature and discussions within the research team, the following research questions were formulated: (1) What is the prevalence of SO among older adults? (2) What assessment tools are currently used to evaluate SO in older adults? (3) What factors influence the occurrence of SO in older adults? and (4) What intervention strategies for SO among older adults have been reported in the existing literature?

### 2.2 Literature search strategy

PubMed, CINAHL, Cochrane Library, Web of Science, China National Knowledge Infrastructure (CNKI), and Wanfang Database were systematically searched using a combination of subject terms and free words. The search period ranged from the foundation of each database to October 3, 2025. For example, the search strategy for PubMed is shown in Box 1. Search strategies were adapted for each database (see Search Strategy Table in Supplementary material).

Box 1 Example of search formula for PubMed.

#1   “aged”[MeSH Terms] OR “elderly”[Title/Abstract] OR “older people”[Title/Abstract] OR “older adult^*^”[Title/Abstract]

#2    “sarcopenic    obesity”[Title/Abstract]    OR    “sarcopenic adiposity”[Title/Abstract] OR “sarcopenia with obesity”[Title/Abstract] OR “obesity with sarcopenia”[Title/Abstract] OR “sarcopenic obese phenotype”[Title/Abstract]

#3 #1 AND #2

### 2.3 Inclusion and exclusion criteria

Inclusion of literature was determined according to the PCC principle.

Inclusion criteria: (1) population (P): older adults with SO, aged ≥60 years; (2) concept (C): studies addressing the prevalence, assessment tools, influencing factors, and intervention strategies related to SO among older adults; (3) context (C): all settings in which older adults with SO receive healthcare services, including healthcare institutions, long-term care facilities, and community settings; (4) study design: cross-sectional studies, cohort studies, randomized controlled trials, and other relevant study designs; and (5) language: studies published in Chinese or English.

Exclusion criteria: studies with incomplete data, duplicate publications, or unavailable full texts.

### 2.4 Study selection

All retrieved records were imported into EndNote (Clarivate Analytics) to remove duplicates. Two researchers who had received formal training in systematic evidence-based methodology independently screened and reviewed the studies according to the predefined inclusion and exclusion criteria, excluding irrelevant articles. Full texts of potentially eligible studies were then assessed in detail, and screening results were compared. Any disagreements were resolved through discussion with a third reviewer to determine final study inclusion.

### 2.5 Collating, summarizing, and reporting the results

A standardized data extraction form was developed to collect information on authors, year of publication, country, sample size, study design, prevalence of SO, assessment tools, influencing factors, intervention strategies, and intervention frequency and duration. Additionally, the diagnostic criteria for sarcopenic obesity reported in each study were compared with the ESPEN–EASO consensus framework, including the coexistence of excess adiposity and sarcopenia domains, the type of muscle parameters assessed (mass, strength, or function), and the obesity indicators used.

## 3 Results

### 3.1 Study selection results

**Figure 1:**
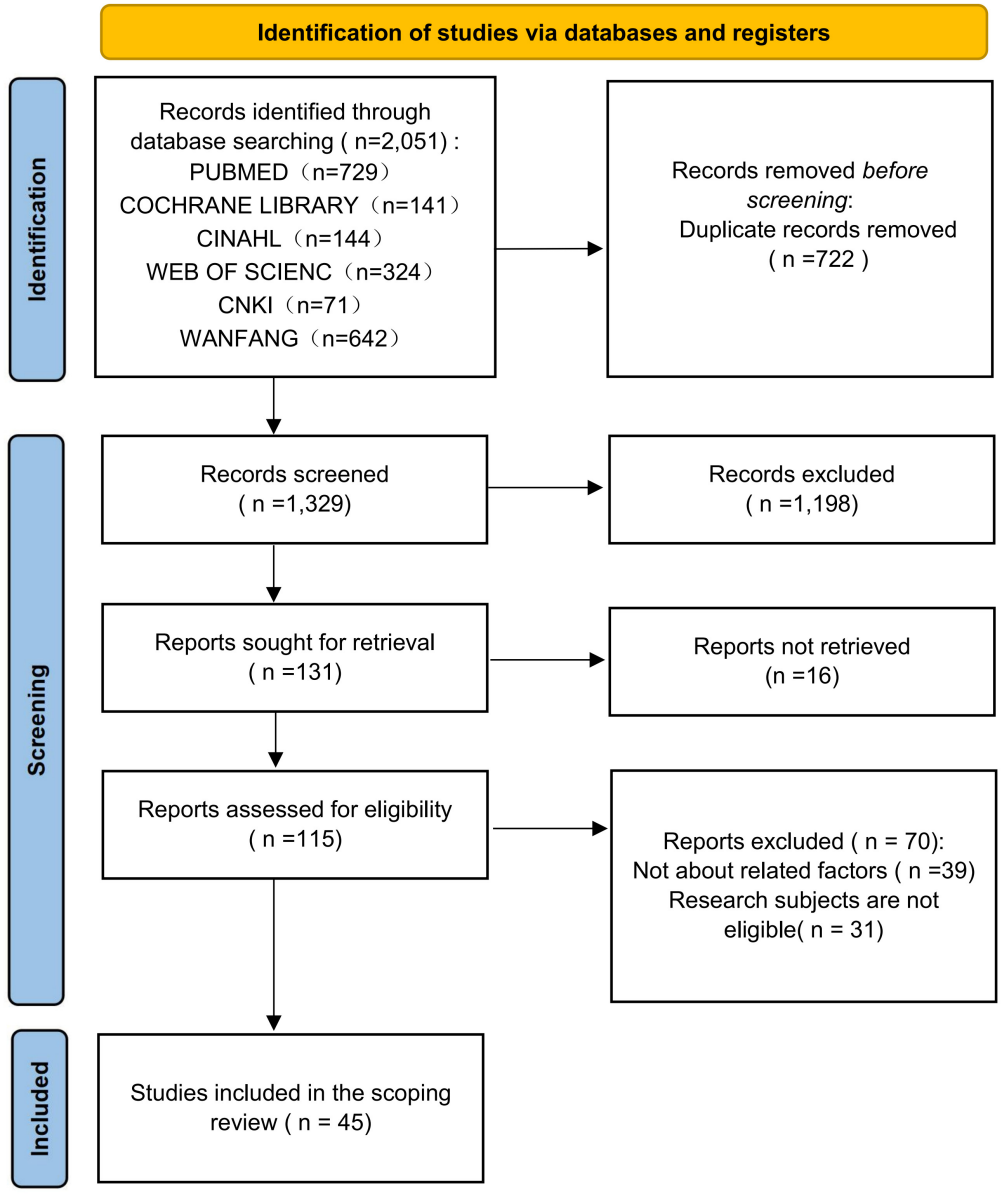
PRISMA diagram of the literature search.

A total of 2,051 records were identified through database searching. After removing duplicates, 1,329 records remained. Following title and abstract screening, 131 articles were retained and independently reviewed in full by two researchers. Of these, 16 were excluded because full texts were unavailable, 39 did not focus on the epidemiology, assessment tools, influencing factors, or interventions related to SO, and 31 involved inappropriate study populations. Ultimately, 45 studies were included in this review. The literature screening process is shown in Figure 1 (PRISMA flowchart).

### 3.2 Characteristics of included studies

A total of 45 studies were ultimately included in this review. The included studies were published between 2016 and 2025, with a marked increase in publication volume observed from 2021 to 2025. Studies originated from the USA (*n* = 2), Spain (*n* = 5), China (*n* = 12), Japan (*n* = 4), South Korea (*n* = 10), Turkey (*n* = 2), Slovenia (*n* = 1), Italy (*n* = 2), Brazil (*n* = 3), Germany (*n* = 1), the Netherlands (*n* = 1), Australia (*n* = 1), and the UK (*n* = 1). Sample sizes ranged from 27 to 8,919 participants. Regarding study design, cross-sectional studies predominated (*n* = 32), cohort studies (*n* = 6), and randomized controlled trials (*n* = 7). The basic characteristics of the included studies are presented in Table 1.

**Table 1:** Basic characteristics of the included studies (*n* = 45).

**Author, year, country**	**Sample size (intervention/control)**	**Study design**	**Prevalence (%)**	**Assessment tools/indices**			**Influencing factors**	**Interventions**
				**Sarcopenia**	**Obesity**	**Stage/SO**		
Xu et al. (11), 2025, China	386	Cross-sectional study	15.80	SMI, HGS, and calf circumference	BMI	–	Activities of daily living	–
Shirakid et al. (12), 2025, Japan	236	Cross-sectional study	9.70	SMM, HGS, and gait speed	BFP	–	Age, masticatory function	–
Polo-Ferrero et al. (13), 2025, Spain	40 (15/12/13)	RCT	8.33	ASMI, HGS, and 5-SST	BMI, BFP	–	Age, chronic inflammation, insulin resistance, physical activity level, and nutritional intake	Exercise interventions
Pérez-Ros et al. (14), 2025, Spain	145	Cross-sectional study	16.55	Screening: SARC-F	Screening: BMI, WC	Staging/–	–	–
				Diagnosis: SMM/W, HGS, and 5-SST	Diagnosis: FMI			
Lian et al. (15), 2025, China	1,431	Cross-sectional study	12.99	SMM/W, HGS, 5-SST, and time to full tandem standing	BFP, WC, thigh circumference	–/Neck circumference	Age, gender	–
Lee et al. (16), 2025, South Korea	6,202	Prospective cohort study	–	ALMI	WC	–	Eating rate	–
Karakurt et al. (17), 2025, Turkey	573	Cross-sectional study	17.28	Screening: HGS, 5-SST, and SARC-F	Screening: BMI, WC	–/Calf circumference/BMI	Hypertension, CHD	–
				Diagnosis: STAR	Diagnosis: BFP			
Guo et al. (18), 2025, USA	5,607	Cross-sectional study	20.31	ASMI	WC	–	Age, ethnicity	–
Eglseer et al. (19), 2025, Slovenia	387	Cross-sectional study	24.50	ASM, HGS	BFP	–	Age, gender, energy intake, nutritional status, and nursing needs	–
Xu et al. (20), 2025, China	5,268	Retrospective cohort study	7.25	ASM/W	BMI	–	Blood lipid level	–
Montalvão-Sousa et al. (21), 2024, Brazil	223	Cross-sectional study	6.73	Screening: clinical symptoms	Screening: BMI/WC	Staging/–	Age, chronic diseases	–
				Diagnosis: HGS	Diagnosis: BFP, FMI			
Li et al. (22), 2024, China	1,353	Cross-sectional study	13.20	SMM/W, HGS	BFP	–	Age, gender	–
Kim et al. (23), 2024, South Korea	5,458	Cross-sectional study	13.95	HGS	WC	–	Diet quality, protein intake	–
Güner et al. (24), 2024, Turkey	234	Cross-sectional study	34.62	SSM/W, HGS	BMI	–/WCR	–	–
Diago-Galmés et al. (25), 2024, Spain	498	Cross-sectional study	13.60	ASMI, HGS, and SPPB	BMI	–/Sar-Qol	Age, quality of life	–
Danielewicz et al. (26), 2024, Italy	90	Cross-sectional study	29.96	Screening: HGS, 5-SST	Screening: BMI	Staging: Stage II/–	Age, gender	–
				Diagnosis: ALM/W, SMM/W	Diagnosis: BFP			
Benz et al. (27), 2024, Netherlands	5,888	Prospective cohort study	10.21	Screening: HGS	Screening: BMI	–	Age, physical activity level, protein intake, chronic diseases, and insulin resistance	–
				Diagnosis: ALM/W	Diagnosis: BFP			
Yoshimura et al. (28), 2023, Japan	760	Cross-sectional study	4.47	Screening: clinical symptoms	Screening: BMI	–/Phase angle	–	–
				Diagnosis: SMM/W, HGS	Diagnosis: BFP			
Yin et al. (29), 2023, China	60 (30/30)	RCT	5.25	HGS, 5-SST	BMI, WC	–	Age, sedentary lifestyle, nutritional status, insulin resistance, oxidative stress, and psychological factors	Nutritional interventions
Scott et al. (30), 2023, Australia	1,416	Prospective cohort study	9.60	HGS, 5-SST, and ALMI	Screening: BMI, WC	–	Age, education level, physical activity level, chronic diseases, vitamin D levels, and depression	–
					Diagnosis: BFP			
Park et al. (31), 2023, South Korea	2,971	Cross-sectional study	4.44	ASMI	WC	–	Nutritional status, physical activity level	–
Lee et al. (32), 2023, South Korea	2,661	Cross-sectional study	2.52	SMI	WC	–	Coffee intake	–
Kim et al. (33), 2023, South Korea	2,031	Cross-sectional study	13.45	ASMI, HGS, and SPPB	BFP, WHtR	–/WWI	–	–
Kim et al. (34), 2023, South Korea	3,821	Cross-sectional study	–	ASM/W	BMI	–/TyG	Insulin resistance, energy intake, physical activity level, chronic diseases, and chronic inflammation	–
Jang et al. (35), 2023, South Korea	3,690	Cross-sectional study	3.88	HGS	WC	–	Age, education level, household income, smoking, diet quality, and nutritional status	–
Fonfría-Vivas et al. (36), 2023, Spain	95	Cross-sectional study	7.37	Screening: SARC-F	BMI, WC	Staging/–	Quality of life, activities of daily living, and nutritional status	–
				Diagnosis: SMI, HGS, and 5-SST				
Diago-Galmés et al. (37), 2023, Spain	202	Cross-sectional study	18.81	ASM, HGS, and gait speed	BMI, WC, BFP, and triceps skinfold thickness	–	Age, gender	–
Yoshimura et al. (38), 2022, Japan	760	Retrospective cohort study	4.47	Screening: clinical symptoms	Screening: BMI	Staging/–	History of stroke	–
				Diagnosis: HGS, SMM/W	Diagnosis: BFP			
Yang et al. (39), 2022, China	2,256	Cross-sectional study	6.25	SMI, HGS, gait speed	BFP	–	Sleep duration, ethnicity	–
Mo et al. (40), 2022, China	1,050	Cross-sectional study	5.77	SMI, HGS, and gait speed	BMI, WC, BFP, and visceral fat area	–	Age, gender	–
Lu et al. (41), 2022, China	1,407	Cross-sectional study	9.95	ASMI, HGS, and gait speed	BFP	–	Age, gender, monocyte levels, and sleep duration	–
Du et al. (52), 2022, USA	8,919	Cross-sectional study	–	SMI, HGS	BMI, BFP, and WC	–	Physical activity frequency	–
Lee et al. (53), 2021, South Korea	27 (15/12)	RCT	–	SMI, HGS, and gait speed	BFP	–	Age, malnutrition, chronic inflammation, and lack of physical activity	Exercise interventions
Lee et al. (42), 2021, South Korea	3,828	Cross-sectional study	13.24	ASM, SMI	BMI	–	Total calorie intake, carbohydrate intake	–
Ren et al. (43), 2021, China	696	Cross-sectional study	7.90	ASMI, HGS	BFP	–	Age, protein intake, and basal metabolism	–
Guo et al. (44), 2021, China	140	Cross-sectional study	17.86	ASMI, HGS, gait speed	BFP	–	Age, albumin level	–
Ribeiro Santos et al. (45), 2020, Brazil	211	Prospective cohort study	10.40	ASMI, HGS, and gait speed	BFP	–	Physical activity levels, sedentary behavior	–
Li et al. (54), 2020, China	30 (15/15)	RCT	–	SMI	BFP	–	Age, hormones, and inflammatory factors	Exercise interventions
Wang et al. (55), 2019, China	80 (20/20/20/20)	RCT	–	ASM, HGS	BMI	–	Age, sedentary behavior, and inflammatory factors, and chronic diseases	Exercise interventions
Perna et al. (46), 2017, Italy	639	Cross-sectional study	12.52	SMI, FFMI	BFP	–	Chronic inflammation, high blood sugar, and nutritional status	–
Oh et al. (47), 2017, South Korea	4,452	Cross-sectional study	21.83	ASM/W	BMI	–	Physical activity, protein intake, vitamin D levels, and metabolic factors	–
Moura Santos et al. (48), 2017, Brazil	1,373	Cross-sectional study	4.44	HGS	BMI	–	Weakness, daily living abilities, and financial situation	–
Kemmler et al. (49), 2017, Germany	100 (33/33/34)	RCT	14.21	SMI, HGS	BFP	–	Age, physical activity level, and knee osteoarthritis	Multidisciplinary interventions
Aggio et al. (50), 2016, UK	1,286	Cross-sectional study	4.98	ASM, HGS, and gait speed	WC	–	Physical activity, sedentary time, and strength training	–
Kim et al. (51), 2016, Japan	139 (36/35/34/34)	RCT	25.31	SMI, HGS, and gait speed	BFP	–	Vitamin D levels, leptin levels	Multidisciplinary interventions

RCT, randomized controlled trial; SMI, skeletal muscle index; HGS, handgrip strength; SMM, skeletal muscle mass; ASMI, appendicular skeletal muscle mass index; 5-SST, 5-times sit-to-stand test; SARC-F, Strength, assistance with walking, rising from a chair, climbing stairs, and falls questionnaire; SMM/W, skeletal muscle mass adjusted for weight; ALMI, appendicular lean mass index; STAR, ratio of anterior thigh muscle thickness to body mass index; ASM, appendicular skeletal muscle mass; ASM/W, appendicular skeletal muscle mass adjusted for weight; SSM/W, skeletal muscle mass adjusted for weight; SPPB, Short Physical Performance Battery; ALM/W, appendicular lean mass adjusted for weight; FFMI, fat-free mass index; BMI, body mass index; BFP, body fat percentage; WC, waist circumference; FMI, fat mass index; WHtR, waist-to-height ratio.

### 3.3 Current research status of SO in older adults

Among the 45 included studies, 39 studies (11–15, 17–33, 35–51) reported the prevalence of SO in older adults, ranging from 2.52 to 34.62%. Sixteen studies (18–20, 23, 25, 27, 33, 35, 37, 38, 41–46) reported sex-specific prevalence, with rates ranging from 5.41 to 23.78% in older women and from 3.68 to 28.95% in older men, as detailed in Table 1.

### 3.4 Utilization of assessment tools of SO in older adults

A total of 23 different assessment tools were used across the included studies. Based on the characteristics of the condition, these tools can be categorized into obesity assessment tools, sarcopenia assessment tools, and comprehensive assessment tools for SO. Obesity assessment tools are relatively simple, with commonly used indicators including body mass index (BMI), waist circumference, and body fat percentage. Twelve studies (13–15, 21, 27, 29, 30, 36–38, 40, 52) employed multiple tools to assess obesity, while fat mass index (14, 21), thigh circumference (15), waist-to-height ratio (33), triceps skinfold thickness (37), and visceral fat area (40) were also used as obesity indicators. Sarcopenia assessment tools are relatively complex and often involve multiple dimensions, including muscle mass, muscle strength, and physical function. For muscle mass assessment, the most commonly used indicators reflect skeletal muscle quantity, such as skeletal muscle index, sarcopenia index, and appendicular skeletal muscle mass index. In contrast, calf circumference (11), STAR index (17), and fat-free mass index (46) were also applied. Handgrip strength was the most frequently used measure of muscle strength, and commonly used physical function assessments included the 5-times sit-to-stand test, gait speed test, and Short Physical Performance Battery (SPPB). The SARC-F questionnaire (14, 36) was also used as a screening tool for sarcopenia. For a comprehensive assessment of SO, tools included neck circumference (15), calf circumference/BMI (17), waist-to-calf ratio (24), phase angle (28), waist-to-weight ratio (33), and triglyceride-glucose index (34). Four studies (11, 15, 18, 42) combined key SO measures to develop risk prediction models specifically for older adults, enabling early identification of those at risk. Additionally, nine studies (14, 17, 21, 26–28, 30, 36, 38) distinguished between screening and diagnostic stages, with questionnaires applied during screening and specific measurements or tools used for diagnostic confirmation. Five studies (14, 21, 26, 36, 38) reported SO staging; one of these studies (26) classified all included patients as stage II, indicating the presence of SO-related complications.

### 3.5 Influencing factors of SO in older adults

#### 3.5.1 Sociodemographic factors

Older adults with specific sociodemographic characteristics were more likely to develop SO, including advanced age (12, 13, 15, 18, 19, 21, 22, 25–27, 29, 30, 35, 37, 40, 41, 43, 44, 49, 53–55), female sex (15, 19, 22, 26, 37, 40, 41), ethnicity (18, 39), low educational level (30, 35), low household income (35), and poor economic status (48).

#### 3.5.2 Disease-related factors

Older adults with certain health conditions were at higher risk for SO, including hypertension (17), hyperglycemia (46), hyperlipidemia (20), stroke (38), coronary heart disease (17), frailty (48), multimorbidity (21, 27, 30, 34, 55), chronic inflammation (13, 34, 46, 53), and metabolic factors such as insulin resistance (13, 27, 29, 34, 43, 47). Six studies (29, 41, 44, 51, 54, 55) also reported associations between physiological indicators—such as estrogen levels, leptin levels, monocyte count, inflammatory markers, and albumin levels—and the risk of SO in older adults.

#### 3.5.3 Psychological behavioral factors

Key factors included low physical activity levels (13, 27, 30, 31, 34, 45, 47, 49, 50, 52, 53), moderate or higher physical activity (31, 50) or resistance training (50), activity frequency (52), impaired activities of daily living (11, 36, 48), poor masticatory function or use of dentures (12), fast eating speed (16), quality of life (24, 36), low care needs (19), sedentary behavior (29, 50, 55), sleep duration (39, 41), psychological factors (29, 30), and current or former smoking (35).

#### 3.5.4 Nutritional factors

Major contributors included poor nutritional status (13, 19, 29, 31, 35, 36, 46, 53), inadequate diet quality (23, 35), total energy intake (42), energy (19, 34), carbohydrate intake (42), protein intake (23, 27, 43, 47), nutrient intake (13), insufficient vitamin D (30, 47, 51), and coffee consumption (32).

### 3.6 Interventions for SO in older adults

#### 3.6.1 Exercise interventions

Four studies designed exercise interventions based on participants' muscle and obesity status. Intervention types included resistance training (13), multicomponent training combining aerobic, resistance, and balance exercises (13), resistance exercise (53, 55), aerobic exercise (54, 55), and combined multimodal training (54, 55). The intervention duration ranged from 8 to 32 weeks. All studies reported that exercise interventions effectively improved body composition in older adults with SO by increasing muscle mass and reducing fat mass, and also enhanced bone mineral density. In addition, Lee et al. (53) found that resistance exercise should be progressive, gradually increasing to moderate-to-high intensity, and without continued training, the beneficial effects diminished at 6-month follow-up.

#### 3.6.2 Nutritional interventions

Yin et al. (29) conducted a 15-week randomized controlled trial based on the Health Action Process Approach combined with behavior change techniques, focusing on dietary behavior modification. The intervention included moderate caloric restriction, adequate protein intake, dietary records, and follow-up guidance. Results demonstrated good feasibility and significant reductions in body weight, as well as improvements in diet quality, handgrip strength, gait speed, and waist circumference. However, skeletal muscle mass index did not change significantly.

#### 3.6.3 Multidomain interventions

In a 12-week randomized controlled trial conducted in Japan, Kim et al. (51) applied a multidomain intervention combining exercise training with nutritional supplementation in 139 community-dwelling older adults with SO. The intervention improved individual outcomes such as body fat, knee muscle strength, and vitamin D levels, but did not affect combined indicators of body fat and muscle/physical function. Kemmler et al. (49) conducted a 16-week trial combining whole-body electromyostimulation (WB-EMS) with protein supplementation. The intervention included WB-EMS training plus daily protein intake of 1.7–1.8 g/kg, whereas the control group received protein supplementation alone. Both interventions reduced body fat and increased skeletal muscle mass index, with the combined WB-EMS and protein intervention showing superior improvement in sarcopenia *Z*-scores and increased handgrip strength.

### 3.7 Alignment of included definitions with the ESPEN–EASO consensus

The diagnostic definitions of sarcopenic obesity reported in the included studies were compared with the ESPEN–EASO consensus framework. Overall, substantial heterogeneity was observed in the inclusion of adiposity and sarcopenia domains, the muscle parameters assessed, and the obesity indicators used. The majority of the studies incorporated both adiposity and sarcopenia components; however, the specific diagnostic parameters differed substantially. While some studies assessed both muscle mass and strength or physical performance, others relied solely on muscle mass indices, thereby only partially reflecting the consensus construct. Similarly, obesity was variably defined using body mass index, waist circumference, body fat percentage, or imaging-based adiposity measures.

Among the studies published after the 2022 consensus, only a minority explicitly adopted diagnostic elements consistent with the ESPEN–EASO recommendations. In contrast, many continued to apply earlier operational definitions or single-domain criteria. Specifically, among the 28 post-2022 studies, 5 (17.86%) (14, 21, 26, 36, 38) showed full alignment with the consensus definition, 7 (25.00%) (15, 17, 22, 24, 27, 28, 30) partial alignment, and 16 (57.14%) (11–13, 16, 18–20, 23, 25, 29, 31–35, 37) no alignment. This heterogeneity in diagnostic composition likely contributes to the wide variation in reported prevalence and associated factors across studies. The extent of alignment with the ESPEN–EASO consensus among post-2022 studies is summarized in Table 2.

**Table 2:** Alignment with the ESPEN–EASO consensus among post-2022 studies (*n* = 28).

**Author**	**Year**	**ESPEN–EASO alignment**	**Diagnostic algorithm applied**	**SO staging reported**
Xu et al. (11)	2025	None	No	No
Shirakid et al. (12)	2025	None	No	No
Polo-Ferrero et al. (13)	2025	None	No	No
Pérez-Ros et al. (14)	2025	Full	Yes	Yes
Lian et al. (15)	2025	Partial	No	No
Lee et al. (16)	2025	None	No	No
Karakurt et al. (17)	2025	Partial	Yes	No
Guo et al. (18)	2025	None	No	No
Eglseer et al. (19)	2025	None	No	No
Xu et al. (20)	2025	None	No	No
Montalvão-Sousa et al. (21)	2024	Full	Yes	Yes
Li et al. (22)	2024	Partial	No	No
Kim et al. (23)	2024	None	No	No
Güner et al. (24)	2024	Partial	No	No
Diago-Galmés et al. (25)	2024	None	No	No
Danielewicz et al. (26)	2024	Full	Yes	Yes
Benz et al. (27)	2024	Partial	Yes	No
Yoshimura et al. (28)	2023	Partial	Yes	No
Yin et al. (29)	2023	None	No	No
Scott et al. (30)	2023	Partial	Yes	No
Park et al. (31)	2023	None	No	No
Lee et al. (32)	2023	None	No	No
Kim et al. (33)	2023	None	No	No
Kim et al. (34)	2023	None	No	No
Jang et al. (35)	2023	None	No	No
Fonfría-Vivas et al. (36)	2023	Full	Yes	Yes
Diago-Galmés et al. (37)	2023	None	No	No
Yoshimura et al. (38)	2022	Full	Yes	Yes

Full: explicit implementation of the ESPEN–EASO diagnostic algorithm, including combined sarcopenia and adiposity criteria and staging; Partial: concurrent assessment of sarcopenia and obesity domains consistent with elements of the consensus but without full algorithm or staging; None: pre-consensus operational definitions without alignment to ESPEN–EASO structure.

## 4 Discussion

### 4.1 Assessment tools for SO in older adults require further structured standardization, highlighting the need for localized and specific instruments

The prevalence of SO in older adults varies widely across studies (2.52%−34.62%), which may be attributed to inconsistencies in the assessment tools used. Numerous instruments exist for evaluating obesity and sarcopenia, but the cut-off points and grading criteria differ, and measurement methods vary across cultural contexts, making it difficult to draw reliable conclusions from the current literature. SO is a multidimensional concept that represents the coexistence of sarcopenia and obesity, and appropriate assessment tools are essential for identifying older adults with SO, analyzing influencing factors, and developing targeted interventions. This review found that most current studies still adopt a simple additive approach combining sarcopenia and obesity diagnostic criteria, failing to reflect the holistic and specific nature of SO as a syndrome.

For obesity assessment, BMI and waist circumference are commonly used for initial screening due to their simplicity, but they do not fully capture the complexity of SO (56). Body fat percentage measured by bioelectrical impedance analysis (BIA) provides more precise body composition information but is limited by equipment availability, cost, and standardization requirements. Other measures used in the included studies, such as triceps skinfold thickness, waist-to-height ratio, and visceral fat area, are less commonly applied and generally less accurate than body fat percentage (33, 37, 40).

Regarding sarcopenia assessment, the SARC-F questionnaire and calf circumference are widely used as screening tools, but require further validation for identifying sarcopenia in older adults (11, 14). Objective measures reflecting muscle mass, such as skeletal muscle index, sarcopenia index, and appendicular skeletal muscle mass, allow early detection of overlooked muscle loss and reduce subjective bias, though they rely on specialized equipment and focus solely on muscle quantity, potentially leading to underestimation (17, 33). Handgrip strength is a convenient, low-cost measure of muscle strength, suitable for community and bedside screening, but it only reflects upper-limb function and does not represent overall muscle status (56). Physical function can be assessed using gait speed, the 5-times sit-to-stand test, and the SPPB (15, 33, 39). Gait speed is the preferred measure due to its simplicity and clinical relevance; the 5-times sit-to-stand test is an alternative when space or patient mobility is limited. The SPPB provides a more comprehensive evaluation of physical function, but it is relatively complex, and evidence for its use in the Chinese population is limited (57).

Emerging comprehensive assessment tools for SO, including neck circumference, waist-to-weight ratio, phase angle, and novel SO prediction models, show potential for screening community-dwelling older adults (15), but their usability and accuracy require further validation in diverse populations. Currently, the diagnosis of sarcopenic obesity largely follows the 2022 consensus published by the European Society for ESPEN–EASO, which established a unified definition, a diagnostic pathway (screening, diagnosis, and staging), and recommended assessment domains. However, the majority of the studies have not strictly adhered to this framework; only nine studies (14, 17, 21, 26–28, 30, 36, 38) distinguished between screening and diagnostic stages, and only five studies (14, 21, 26, 36, 38) reported patient staging. Among studies published after the 2022 ESPEN–EASO consensus, only 5 (18%) showed full alignment with the consensus definition, 7 (25%) partial alignment, and 16 (57%) no alignment. The limited uptake of the ESPEN–EASO framework may reflect differences in available measurement techniques, historical definitions, and ongoing transitions in diagnostic practice. However, inconsistent operationalization of sarcopenic obesity directly affects comparability of prevalence estimates and risk associations across populations.

In future clinical research, the ESPEN–EASO consensus should be more widely adopted to reduce definitional heterogeneity and improve the interpretability of findings. At the same time, researchers should integrate multidimensional indicators of muscle mass, strength, function, and fat distribution, leveraging technologies such as bioelectrical impedance and machine learning to develop more accurate, convenient, and primary care applicable prediction tools for older adults with SO. These tools should be culturally adapted, with a focus on determining optimal screening cut-off points for clinical practice to enable timely identification and preventive management, and to guide healthcare professionals in targeting interventions effectively to slow the progression of SO.

### 4.2 Influencing factors of SO in older adults are complex and multifactorial and warrant further identification and attention

#### 4.2.1 Sociodemographic factors

There is general agreement that educational attainment, household income, and economic status are associated with SO in older adults; however, the relationships between age, sex, and SO remain controversial. Several studies have reported that the risk of SO increases with advancing age (12, 15, 18, 19, 21, 22, 25–27, 30, 35, 40, 41, 43, 44), whereas one study found no association between age and SO (37), possibly due to differences in diagnostic criteria and a relatively narrow age range of participants. Regarding sex differences, some studies indicated that older men have a higher risk of SO than older women (15, 19, 22, 41), while others reported opposite findings (26, 40). One study also suggested no significant association between sex and SO (37), which may be related to sex distribution, regional differences, and unmeasured confounding factors. Variations in ethnicity, cultural background, and lifestyle may further influence perceptions of health and behaviors, thereby affecting the occurrence of SO. For sociodemographic factors with inconsistent findings, large-scale, multicenter studies with adequate control of confounders are needed to clarify these associations.

#### 4.2.2 Disease-related factors

Evidence indicates that hypertension, hyperglycemia, and dyslipidemia are common disease-related factors associated with SO in older adults. These metabolic abnormalities may inhibit muscle protein synthesis, accelerate muscle loss, and promote fat accumulation (34, 47). Chronic conditions such as coronary heart disease may indirectly contribute to muscle loss and subsequently increase SO risk (17). Frailty, chronic inflammation, and insulin resistance are also important contributors. Reduced muscle mass and impaired muscle function are core features of frailty, which further limit physical activity in older adults (48). SO is often characterized by chronic low-grade inflammation, a key trigger of insulin resistance. Inflammatory mediators disrupt lipid metabolism by inhibiting lipolysis and promoting ectopic fat deposition (46), while insulin resistance negatively affects muscle protein synthesis and degradation, thereby exacerbating muscle loss (34). In addition, estrogen levels, leptin levels, and monocyte counts have been reported to be associated with SO risk, potentially increasing susceptibility through inflammatory pathways, altered fat distribution, and nutritional status (41, 43, 44, 51). Future large-sample, longitudinal, prospective studies are needed to elucidate underlying mechanisms further and inform the development of individualized interventions.

#### 4.2.3 Psychological behavioral factors

The majority of the studies consistently identify insufficient physical activity as a core factor contributing to SO in older adults. Moderate-to-vigorous physical activity or strength training performed at least 3 times per week has been shown to reduce SO risk, as adequate activity promotes muscle protein synthesis through mechanical loading and reduces visceral fat accumulation. In contrast, physical inactivity accelerates disuse-related muscle loss and fat gain (31, 50, 52). Poor activities of daily living (11, 36, 48), lower quality of life (25), impaired chewing function (12), rapid eating speed, and low care needs (16, 19) may increase SO risk through multiple pathways, including reduced mobility, inadequate intake of high-quality protein, and unrecognized nutritional deficiencies. Findings regarding sedentary behavior and sleep duration are inconsistent. Prolonged sedentary time of 30–60 min per day has been associated with increased SO risk, although one study reported no association, possibly due to unclear definitions of sedentary behavior or protective sample characteristics (45). Sleep duration of 9 h or longer has been linked to higher SO risk (39, 41), potentially due to reduced activity time and sleep-related metabolic disturbances. Psychological factors, including low self-efficacy and poor adherence, may also contribute to SO development (29, 30). Therefore, comprehensive assessment of physical function, early monitoring of physical activity, functional capacity, sleep patterns, and psychological health are essential to inform the development of safe and effective intervention strategies.

#### 4.2.4 Nutritional factors

Poor nutritional status is a key determinant of SO in older adults. Inadequate diet quality and insufficient total energy intake may trigger muscle protein breakdown to supply energy and reduce fat oxidation efficiency (23, 34, 42). Insufficient protein intake directly suppresses muscle synthesis (27, 43), while imbalanced carbohydrate and fat intake disrupts metabolic regulation, it further promotes muscle loss and fat accumulation. Vitamin D deficiency impairs muscle function and increases the risk of SO risk (30, 47, 51). One study reported a higher risk of SO among older adults consuming less than one cup of coffee per day compared with those consuming more than three cups (32). This finding should be interpreted with caution, as other dietary and physical activity behaviors may confound coffee consumption, and key variables were not fully controlled. Moreover, this result was derived from a Korean population, and its generalizability to older adults in China requires further validation. Overall, existing studies largely rely on self-reported questionnaires to assess nutritional intake and have not adequately examined synergistic effects among nutrients. More precise assessment methods and robust validation studies are needed to support evidence-based nutritional interventions.

### 4.3 Interventions for SO in older adults require further optimization and development

Among the 40 studies included in this review, only seven were interventional studies targeting older adults with SO, indicating a clear lack of intervention research in this population. Small sample sizes, insufficient consideration of individual heterogeneity among older adults, and limited long-term follow-up characterize existing studies. Evidence related to exercise-based interventions is relatively more established (58), whereas studies focusing on dietary and nutritional interventions, as well as on multidomain combined approaches, remain scarce. Notably, no studies addressing psychological interventions for older adults with SO were identified.

Future research should more fully account for individual differences among older adults with SO, expand sample sizes, and emphasize the long-term effects of interventions to enhance the generalizability and representativeness of findings. In addition, multidimensional and integrated intervention strategies should be developed, with greater attention to psychological wellbeing and strengthened multidisciplinary collaboration, to improve intervention safety and long-term adherence.

## 5 Conclusion

This review synthesized existing evidence on the prevalence, assessment tools, influencing factors, and interventions related to SO in older adults. However, the analysis was limited to a descriptive synthesis of the included studies, without a formal quality appraisal, which may have introduced bias and influenced the robustness of the conclusions. Current evidence indicates substantial variability in the reported prevalence of SO among older adults. In terms of assessment, a unified and standardized evaluation framework is lacking, and existing diagnostic cut-off values are not sufficiently specific. Adoption of the ESPEN–EASO consensus in recent studies remains incomplete, contributing to persistent heterogeneity in sarcopenic obesity definitions and reported prevalence. Future efforts should integrate multidimensional indicators and promote broader adoption of the ESPEN–EASO consensus definition and diagnostic framework to improve consistency and comparability across studies, thereby supporting the development of more accurate, convenient, and culturally appropriate predictive assessment tools for older adults. Such tools can assist healthcare professionals in the early identification of sarcopenic obesity and in selecting appropriate intervention targets.

In addition, greater attention should be paid to the complex, interacting factors influencing SO in older adults, with clarification of the synergistic effects of different determinants. Through long-term observation of the change trajectory, in-depth study of its pathogenesis, design and carry out multidisciplinary and multidomain intervention measures, so as to delay the progression of older adult SO patients, improve their quality of life, and promote healthy aging.

## Data Availability

The original contributions presented in the study are included in the article/Supplementary material, further inquiries can be directed to the corresponding author.
