# Supplementary material for: Sarcopenic obesity in older adults: a scoping review of prevalence, assessment tools, associated factors, and interventions
Source: Front Public Health. 2026 Mar 24;14:1792543. doi: 10.3389/fpubh.2026.1792543 (PMC13054717; doi:10.3389/fpubh.2026.1792543)
Supplement: Supplementary file 1 [file Table_1.docx]

***Supplementary Material***

**Search strategy**

| Total (Searched by 2025.10, Items found: 2,051 ) | |
| --- | --- |
| 1. Pubmed (Items found: 729) | |
| #1 | “aged”[MeSH Terms] OR “elderly”[Title/Abstract] OR “older people”[Title/ Abstract] OR “older adult*”[Title/Abstract] |
| #2 | “sarcopenic obesity”[Title/Abstract] OR “sarcopenic adiposity”[Title/Abstract] OR “sarcopenia with obesity”[Title/Abstract] OR “obesity with sarcopenia”[Title/ Abstract] OR “sarcopenic obese phenotype”[Title/Abstract] |
| #3 | #1 AND #2 |
| 1. Cochrane Library (Items found: 141) | |
| #1 | MeSH descriptor: [Aged] explode all trees |
| #2 | (elderly OR “older people” OR “older adult*” ):ti,ab,kw |
| #3 | #1 OR #2 |
| #4 | MeSH descriptor: [Sarcopenia] explode all trees |
| #5 | MeSH descriptor: [Obesity] explode all trees |
| #6 | (“arcopenic obesity” OR “arcopenic adiposity” OR “sarcopenia with obesity” OR “obesity with sarcopenia” OR “sarcopenic obese phenotype”):ti,ab,kw |
| #7 | #4 AND #5 |
| #8 | #6 OR #7 |
| #9 | #3 AND #8 |
| 1. CINAHL (Items found: 144) | |
| S1 | TI aged OR TI elderly OR TI “older people” OR TI “older adult*” |
| S2 | AB aged OR AB elderly OR AB “older people” OR AB “older adult*” |
| S3 | S1 OR S2 |
| S4 | TI “sarcopenic obesity” OR TI “sarcopenic adiposity” OR TI “sarcopenia with obesity” OR TI “obesity with sarcopenia” OR TI “sarcopenic obese phenotype” |
| S5 | AB “sarcopenic obesity” OR AB “sarcopenic adiposity” OR AB “sarcopenia with obesity” OR AB “obesity with sarcopenia” OR AB “sarcopenic obese phenotype” |
| S6 | S4 OR S5 |
| S7 | S3 AND S6 |
| 1. Web of science (Items found: 324) | |
| S1 | TS=(aged OR elderly OR “older people” OR “older adult*”) |
| S2 | TS=(“sarcopenic obesity” OR “sarcopenic adiposity” OR “sarcopenia with obesity” OR “obesity with sarcopenia” OR “sarcopenic obese phenotype”) |
| S3 | S1 AND S2 |
| 1. China National Knowledge Infrastructure (CNKI) (Items found: 71 ) | |
| #1 | SU%=老年 OR 老年人 OR 老年患者 |
| #2 | SU%=肌少性肥胖 OR 少肌性肥胖 OR 肥胖性肌少症 OR 肌肉减少性肥胖 OR 肥胖性肌肉减少症 OR 肌肉减少症性肥胖 |
| #3 | #1 AND #2 |
| 1. Wanfang Database (Items found: 642) | |
| #1 | 主题：老年 OR 老年人 OR 老年患者 |
| #2 | 主题：肌少性肥胖 OR 少肌性肥胖 OR 肥胖性肌少症 OR 肌肉减少性肥胖 OR 肥胖性肌肉减少症 OR 肌肉减少症性肥胖 |
| #3 | #1 AND #2 |
